# Comprehensive Evaluation of Clinical Application of Balanced Compound Amino Acid Injection

**DOI:** 10.3389/fnut.2022.880256

**Published:** 2022-06-02

**Authors:** Yingqin Shi, Hai Song, Jinyan Liu, Jie Lin, Lingzhi Fang

**Affiliations:** ^1^Department of Pharmacy, Hebei General Hospital, Shijiazhuang, China; ^2^Department of Science and Education, Tangshan People’s Hospital, Tangshan, China; ^3^Department of Pharmacy, The Second Hospital of Tangshan, Tangshan, China

**Keywords:** balanced compound amino acids for injection, clinical nutrition, economy, safety, nutritional quality

## Abstract

**Background:**

To provide a reference for hospital drug selection and rational clinical drug selection based on the evaluation of the safety, nutritional quality, and economy of 27 manufacturers of five varieties (18AA, 18AA-I, 18AA-II, 18AA-IV, 18AA-V) of balanced compound amino acids for injection and (18AA-II_original research_).

**Methods:**

The safety of compound amino acids for injection was evaluated by comparing the antioxidant sulfite contents. Based on the amino acid scoring standard mode and the whole egg protein mode as proposed by the Food and Agriculture Organization of the United Nations/World Health Organization (FAO/WHO) in 1973, we compared the formula. The first limiting amino acid content and the comprehensive quality of the total essential amino acid (EAA) contents of the six formulations were studied. The price/content ratio was used to evaluate their economy.

**Results:**

Similar variety produced by different manufacturers have the same formula and contents of balanced compound amino acids for injection. Safety: 18AA-II_original research_ and 18AA-II had the lowest sulfite content. Compared with 18AA-II_original research_, the sulfite content of 18AA-I, 18AA, 18AA-V, and 18AA-IV were higher (10 times, 16.67 times, 16.67 times, and 33.33 times, respectively). The lower the sulfite content, the safer the product. Nutritional quality: The proportions of amino acids in the five varieties of compound amino acid injection were all suitable. The order of the first limiting amino acids for the formulations was 18AA-II_original research_ = 18AA-II>18AA >18AA-I = 18AA-IV>18AA-V. The order of the EAA values for the formulations was 18AA-II_original research_ = 18AA-II>18AA>18AA-I > 18AA-IV > 18AA-V. The overall effectiveness order was 18AA-II_original research_ = 18AA-II>18AA > 18AA-I>18AA-IV>18AA-V. Economy: Among the 27 manufacturers, 12 manufacturers had a price/content ratio higher than that of 18AA-II original research manufacturers, and 15 manufacturers had a price/content ratio lower than original research manufacturers.

**Conclusion:**

Through its security, effectiveness, and economy of the comprehensive research, we recommended 18AA-II and 18AA-IIoriginal research with high safety, efficacy, and reasonable price as the first choice. 18AA and 18AA-I with better safety and reasonable price, secondary recommendation. 18AA-IV or 18AA-V with poor safety, efficacy, and economy are not recommended.

## Background

Amino acids (AAs) are the basic components of protein (1). For patients who are unable to tolerate or absorb amino acids, parenteral nutrition is very important for the synthesis of human proteins (2, 3). Twenty naturally occurring amino acids are required for the synthesis of human proteins (4). Patients with normal liver and kidney functions are required to eat a diet that is balanced in amino acids. This requires eight essential amino acids (EAA), two semi-essential amino acids, and non-essential amino acids (NEAA) represented in the diet at an EAA/NEAA ratio of approximately 1:1–3 (5). At present, there are many formulations of balanced amino acids for injection in China, produced by numerous manufacturers, with variations in the content and price. The total AA content, as well as the contents of the different EAAs, are related to the safety, effectiveness, and economy of these clinical medications. 18AA-II_original research_ is a balanced amino acid developed by Fresenius Kabi and was listed in Sweden and approved by the State Food and Drug Administration (FDA) in 1983 (6). It was officially listed in China in 1998. Its function is to supplement amino acids in patients with insufficient protein intake and absorption disorders and to improve the nutritional status of patients after surgery. To provide a reference for hospital drug selection and rational clinical drug selection, we compared the safety, nutritional quality, and economy of five varieties of balanced compound amino acids injection produced by 27 manufacturers in our province with 18AA-II_original research_.

## Materials and Methods

### Data Sources

The sulfite and amino acid contents of the five formulations and 18AA-II_original research_ were obtained from the latest version of the drug manual approved by the National Drug Administration (NMPA). The price of each bottle of compound amino acid injection was obtained from http://60.205.165.231/deal/purchase/toaction, Hebei Pharmaceuticals centralized purchase platform.

### Safety Evaluation

Drug safety refers to the degree of influence on the safety of human life after administration according to the specified indications in terms of usage and dosage and is closely related to adverse drug reactions, which can occur during or after drug use (7). Drug safety is evaluated in toxicology and pharmacology research before listing and adverse drug reactions (ADR), adverse drug events (ADE), government management (product recall, withdrawal, warning, instruction modifications, quality spot-checks), and similar comparisons after listing.

The adverse reactions, contraindications, careful use, special populations (elderly, children, pregnant and lactating women), drug interactions, and precautions listed in the instructions for use of compound amino acids for injection produced by different manufacturers are the same. ADR and ADE are mainly evaluated after the drug reaches the market.

Yu and Li reported that the common clinical adverse reactions of compound amino acids for injection were thrombophlebitis, pain, and subcutaneous edema during infusion, nausea, vomiting, chest distress, palpitation, chills, fever, and headache. Furthermore, the concentration of amino acids, infusion mode (central vein or peripheral infusion), infusion speed, infusion site, single bottle amino acid infusion, or complete gastrointestinal operation. Higher concentrations of amino acids and faster infusion speed are related to a higher incidence of adverse reactions, which is consistent with the amino acid specification. The adverse reactions can be reduced or avoided by slowing down the intravenous infusion speed, changing the infusion mode or the infusion site, and reducing the total liquid amino acid concentration, which is generally not related to the drug of that manufacturer (8, 9).

Compound amino acid injections contain the essential amino acid tryptophan, which is easily oxidized and leads to a decrease in the content. Therefore, the antioxidant sulfite should be added to maintain the stability of the preparation. There are two main hazards to sulfite in humans: inducing allergic reactions (especially in asthmatic patients, and manifested as rash, pruritus, and anaphylactic shock in severe cases) and damaging tissues and organs. Sulfite is an important factor leading to ADR/ADE of compound amino acid injection. Li Wenwu and Yan Xuelian reported risk signal monitoring of ADR/ADE in the clinical manifestations after injection of compound amino acids in two ways of ror and X^2^ test: the allergic reaction was the first (8, 10, 11).

The higher the content of sodium sulfite, the more likely the adverse reactions will occur (12–14). Its safety was evaluated by comparing sulfite content.

### Nutritional Quality Evaluation

In the evaluation of a drug treatment program, the effect is an important goal. The purpose of amino acid supplementation in parenteral nutrition is to synthesize human proteins. The more similar the amino acid is to the human body’s amino acid pattern, the better the protein that can be synthesized, and the higher the nutritional value. The nutritional value of amino acids for injection depends mainly on the content, type, and proportional composition of the EAAs. According to the amino acid scoring standard mode and whole egg protein mode recommended by the FAO/WHO in 1973, we evaluated the nutritional value of 18AA-II_original research_ and five formulations of compound amino acids for injection, the first limiting amino acid, and the total EAA quality.

In the calculation of EAA, cysteine is converted from methionine, and tyrosine is similar to phenylalanine. *In vivo*, phenylalanine is converted from tyrosine. Therefore, cysteine and methionine are combined and phenylalanine and tyrosine are combined.

#### Formula Evaluation of Compound Amino Acids for Injection

The FAO/WHO proposed that EAA should account for more than 40% of the AA in an ideal protein, with a ratio of the nitrogen content of EAA/AA value (E/T value) greater than 2.5. This is an important reference index on which to investigate formulations of compound amino acids for injection (15).

Percentage of EAA in total amino acids


(EAA/AA)×100%

Nitrogen content ratio of EAA and AA (E/T value)


E/T=EAA/(AA×16%)⁢(16)

Evaluation of the first limiting amino acid of formulations of compound amino acids for injection.

Among the evaluated proteins, the contents of one or more EAAs were relatively low, which leads to the waste of other EAAs. EAAs present at low levels are known as restricted amino acids, and the one with the lowest content is the first restricted amino acid. The lower the content of the first limiting amino acid, the lower the nutritional value of the amino acid, leading to a limitation of the synthesis of proteins in vivo is limited, and reduced nutritional quality of the formulation.

Chemical score (CS) using egg protein as the standard model (17). CS is used to evaluate the closeness of the relative content of a certain EAA in the total EAA of the protein to that of the corresponding EAA in the standard egg protein. The closer the CS is to 1, the closer the composition is to the standard egg protein, and the higher the nutritional value.


CS=AA/AAEGG

AA: the content of amino acid in the test sample,%; AAEGG: the content of the same amino acid in the whole egg protein,%;

Amino acid score (AAS), the standard mode of FAO/WHO (18). AAS is the percentage of the relative content of a certain EAA in the total EAA of the evaluated protein in the corresponding amino acids according to the WFO/FAO scoring mode. The closer the AAS value is to 1, the closer the amino acid composition is to the WFO/FAO scoring mode, the higher the protein value, and the higher the amino acid nutritional quality.


AAS=AA/AAFAO/WHO

AA: the content of amino acids in the test sample,%; AA FAO/WHO: the content of amino acids in the WFO/FAO scoring mode,%;

Comprehensive quality evaluation of total EAAs in the formulation of compound amino acids for injection.

In the evaluation of protein quality, the total EAA should be considered in addition to the content of a certain EAA.

The essential amino acid index (EAAI) is the standard model of egg protein (19, 20).

The EAAI is used to reflect the proportion of all EAAs in the evaluated protein compared to that of all EAAs in the egg protein. It is used to evaluate the comprehensive quality of a certain protein. The closer the EAAI value is to 100, the closer the EAA composition of the evaluated protein is that of egg protein, the greater the ability of the amino acid to be used for protein synthesis, and the higher the nutritional quality of the formulation.


EAAI=(100⁢A/AE×100⁢B×BE×⁢100⁢C/C⁢E⁢…⁢1/N)

N: the number of essential amino acids; a, B, C, –: the content of essential amino acids in the detected amino acids, %; AE, be, CE –: the content of essential amino acids in the whole egg protein, %.

The score of the ratio coefficient of amino acid (SRCAA) based on FAO/WHO as the standard mode (19, 20).

The SRCAA is calculated based on the ratio of amino acids (RAA) and the ratio coefficient of an amino acid (RCAA). The SRCAA of EAA in the evaluated protein is based on the EAA model of the FAO/WHO. The closer the SRCAA is to 100, the greater the ability of the amino acid to be used for protein synthesis, the higher the nutritional quality and the higher the nutritional value.


/gprotein)/correspondingEAAcontentinFAO/WHO

formula⁢(mg/g⁢protein)

RCAA=averageofRAA/RAA

SRCAA=100-CV×100

CV: the coefficient of variation of RCAA; CV = standard deviation/mean.

Evaluation of the closeness of the total EAA and standard protein (whole egg protein or FAO/WHO model) in the formulation of the compound amino acids for injection.

According to Lan and Apos’s distance method (18), the closeness μ (a, UI) of UI and standard protein A (whole egg protein or FAO/WHO mode) of the evaluation object is calculated according to formula 5: RAA = EAA content of protein to be evaluated (mg).


$$\mu \,\left(a,\,u_{i}\right)=1-0.09\,\sum_{k=1}^{\infty }\frac{\left|a_{k}-u_{i\,k}\right|}{a_{k}+u_{i\,k}}$$

*a*_*k*_ (k = 1,2…8) is the k*^th^* EAA content of standard protein (whole egg protein or FAO/WHO mode) a (mg ⋅ g); uik (k = 1,28) is the content of EAA (mg ⋅ g) of K (EAA corresponding to standard protein) of the i*^th^* evaluated object.

The closeness value reflects the closeness between the protein quality of the evaluated object and the standard protein (whole egg protein or FAO/WHO mode). The closer the value is to 1, the higher the closeness is to the standard protein, the higher the nutritional value, and the higher the amino acid availability.

### Economic Evaluation

Parenteral nutrition consists of three macronutrients: fat emulsion, glucose, and amino acids, of which fat emulsion and glucose are used to provide calories (1 g fat provides 9 kcal heat; 1 g glucose provides 4 kcal heat), and are designated non-protein heat sources. Amino acids are used primarily to synthesize proteins and are not for energy production (protein heat source). To ensure that patients consume sufficient amino acids to synthesize human proteins without being consumed as heat, it is necessary to provide sufficient non-protein heat resources and amino acids. The amino acid requirements of individuals are age and disease-dependent, although the general administration range is 0.6-2.0 (g⋅kg^–1^⋅d^–1^). In parenteral nutrition prescriptions, the proportion and quantity of three macronutrients should be considered. The liquid quantity is the sum of the liquid quantity of nutrition preparation and the liquid quantity of the treatment drug. Adult patients need 30–40 ml⋅kg^–1^⋅d^–1^ liquid every day. Patients with abnormal liver, kidney, and heart functions and the elderly require strict control of the total liquid quantity. In cases where liquids are limited, greater quantities of AAs are beneficial for the synthesis of albumin (13).

In Hebei province, there are 28 manufacturers of five formulations of compound amino acids for injection (plus 18AA-II_original research_) for clinical use. The amino acid contents and prices of these formulations differ. In this study, we compared the price and content ratio (price/content) of each bottle of compound amino acids for injection from the 28 manufacturers. Lower price/content ratios correspond to lower medical costs to achieve the same treatment, which is an economic advantage for patients and the country. Furthermore, a comparison of the price/content ratio can provide a reference for drug purchase decisions.

## Results

The sulfite contents of the six different formulations of the compound amino acids for injection are shown in Table 1.

**TABLE 1:** Sulfite contents of the five formulations of the 27 manufacturers and 18AA-II_original research_.

	18AA	18AA -1	18AA-II_(Original research)_	18AA-II (8.5%)	18AA-IV	18AA - V
Manufacturer(number)	16	1		3	2	5
Sulfite content (g)	0.5	0.3	0.03	0.03	1	0.5
18AA-II_(Original research)_ times	16.67	10		the same	33.33	16.67

Table 1 shows that the minimum sulfite content of 18AA-II_original research_ was 0.03g. The sulfite content of 18AA-II sulfite was equal to that of 18AA-II_original research_. The maximum sulfite content of 18AA-IV was 1 g, which was 33.33 times that of 18AA-II. The sequence of sulfite contents from high to low was 18AA-IV > 18AA = 18AA-V > 18AA-I > 18AA-II = 18AA-II_original research._18AA-II and 18AA-II_original research_ are the least likely to induce anaphylaxis and damage to tissues and organs.

Evaluation of formula rationality of 18AA-II and five formulations of compound amino acids for injection_original research_.

The composition of 18AA-II_original research_ and five formulations of amino acids for injection (from NMPA), EAA as a percentage of the total AA and the E/T ratio are shown in Table 2.

**TABLE 2:** Composition, EAA as a percentage of the total AA and the E/T ratio of 18AA-II_original research_ and five formulations of amino acids for injection.

	18AA	18AA-I	18AA- II _(original research)_	18AA-II (8.5%)	18AA-IV	18AA-V
Lysine	4.30	4.90	9.50	9.50	4.13	3.33
Valine	3.60	4.30	5.50	5.50	1.50	1.36
Threonine	2.50	3.00	4.20	4.20	2.17	1.97
Isoleucine	3.52	3.90	4.20	4.20	1.87	1.70
Tryptophan	0.90	1.00	1.40	1.40	0.52	0.39
Methionine + Cysteine	2.25	2.05	4.20	4.20	1.65	1.50
Leucine	4.90	5.30	5.90	5.90	4.17	3.79
Phenylalanine + Tyrosine	5.58	6.00	6.10	6.10	3.23	2.94
Sigma EAA	27.55	30.45	41.00	41.00	19.24	16.98
Cystine	0.10		0.40	0.40		
Arginine	5.00	3.30	8.40	8.40	2.63	2.89
Alanine	2.00	3.00	12.20	12.20	2.07	1.88
Aspartic acid	2.50	4.10	2.50	2.50	1.27	1.15
Histidine	2.50	2.40	5.00	5.00	2.00	2.46
Serine	1.00	7.50	3.40	3.40	0.73	0.67
Glutamate	0.75	9.00	4.20	4.20	2.17	1.97
Proline	1.00	8.10	5.00	5.00	1.10	1.00
Glycine	7.60	2.10	5.90	5.90	3.57	3.24
Sodium bisulfite	0.50	0.30	0.03	0.03	1.00	0.50
Sigma AA	50.00	69.95	87.65	87.65	34.78	32.24
Sigma EAA/Sigma AA	55.10	43.53	46.78	46.78	55.32	52.67
E/T	3.34	2.64	2.84	2.84	3.36	3.20

Table 2 shows that the percentage of EAA in the total AA of 18AA-II_original research_ and five formulations of amino acids for injection exceeded 40%, and the E/T ratio was higher than 2.5. Thus, the formulation proportion is acceptable.

To facilitate a unified evaluation, the amount of each EAA prescription g ⋅ 1,000 ml^–1^ was converted into the content of each amino acid in AA mg ⋅ G protein^–1^. The results are shown in Table 3.

**TABLE 3:** EAA of compound amino acids for injection.

	Whole egg protein	18AA	18AA-I	18AA-II_(original research)_	18AA-II (8.5%)	18AA-IV	18AA-V	FAO/WHO
Lysine	70.00	86.00	70.10	108.50	108.50	118.70	103.30	55.00
Valine	66.00	72.00	61.50	62.70	62.70	43.10	42.20	50.00
Threonine	47.00	50.00	42.90	47.90	47.90	62.40	61.10	40.00
Isoleucine	54.00	70.40	55.80	47.90	47.90	53.80	52.70	40.00
Tryptophan	17.00	18.00	14.30	16.00	16.00	15.00	12.10	10.00
Methionine + cysteine	57.00	45.00	29.30	47.90	47.90	47.40	46.50	35.00
Leucine	86.00	98.00	75.80	67.30	67.30	119.90	117.60	70.00
Phenylalanine + tyrosine	93.00	111.60	85.80	69.60	69.60	92.90	91.20	60.00
Sigma EAA	490.00	551.00	435.50	467.80	467.80	553.20	526.70	360.00

*Evaluation of the first limiting amino acid of 18AA-II_original research_ and five formulations of amino acids for injection.*

The CS and ASS evaluation results are shown in Table 4.

**TABLE 4:** The CS and ASS of 18AA-II_original research_ and five formulations of amino acids for injection.

	18AA		18AA-I		18AA-II_(original research)_		18AA- II (8.5%)		18AA-IV		18AA-V	
	CS	AAS	CS	AAS	CS	AAS	CS	AAS	CS	AAS	CS	AAS
Lysine	1.09	1.02	1.13	1.05	1.62	1.52	1.62	1.52	1.50	1.40	1.37	1.28
Valine	0.97	0.94	1.05	1.02	1.00	0.97	1.00	0.97	0.58	0.56	0.59	0.58
Threonine	0.95	0.82	1.03	0.89	1.07	0.92	1.07	0.92	1.18	1.02	1.21	1.04
Isoleucine	1.16	1.15	1.16	1.15	0.93	0.92	0.93	0.92	0.88	0.88	0.91	0.90
Tryptophan	0.94	1.18	0.95	1.18	0.99	1.23	0.99	1.23	0.78	0.98	0.66	0.83
Methionine + Cysteine	0.70	0.84	0.58	0.69	0.88	1.05	0.88	0.88	0.74	0.88	0.76	0.91
Leucine	1.01	0.91	0.99	0.90	0.82	0.74	0.82	0.82	1.23	1.11	1.27	1.15
Phenylalanine + Tyrosine	1.07	1.22	1.04	1.18	0.78	0.89	0.78	0.78	0.88	1.01	0.91	1.04

Table 4 shows that the first limiting amino acid was different from the standard protein mode.

The CS value of the first limiting amino acids of 18AA-II_original research_ and 18AA-II were the highest at 0.78, which was closest to the egg protein standard mode. The CS value of the first limiting amino acid of 18AA-I, 18AA-IV, and 18AA-V was the lowest at 0.58, 0.58, and 0.59, respectively, which was far lower than that of the egg protein standard mode.

The order of the first limiting amino acids for the formulations was: 18AA-II_original research_ = 18AA-II > 18AA > 18AA-I = 18AA-IV > 18AA-V.

The AAS value of the first limiting amino acids of 18AA at 0.82, which was closest to the FAO/WHO protein standard mode. The AAS value of the first limiting amino acid of18AA-IV was the lowest at 0.56, which was far lower than that of the FAO/WHO protein standard mode.

The order of the first limiting amino acids for the formulations was: 18AA > 18AA-II_original research_ = 18AA-II > 18AA-I ≥ 18AA-V > 18AA-IV.

Comprehensive quality evaluation results of total EAA content of 18AA-II_original research_ and five formulations of amino acids for injection.

Table 5 shows that compared with egg protein, the highest EAAI value was obtained for 18AA-II_original research_ and 18AA-II at 98.55, and the lowest value was obtained for 18AA-V at 92.15. The order of the EAAI values for the formulations was 18AA-II_original research_ = 18AA-II > 18AA > 18AA-I > 18AA-IV > 18AA-V.

**TABLE 5:** EAAI, SRCAA, and closeness of 18AA-II_original research_ and five formulations of amino acids for injection to the standard protein.

	18AA	18AA-I	18AA-II_(original research)_	18AA-II (8.5%)	18AA- IV	18AA-V
_*EAAI*_	97.7	97.1	98.55	98.55	93.21	92.15
SRCAA	84.76	82.76	76.69	76.69	75.69	77.72
Egg closeness	0.94	0.94	0.94	0.94	0.92	0.91
FAO/who closeness	0.85	0.91	0.90	0.90	0.85	0.87

Table 5 shows that compared with FAO/WHO protein standard mode, the highest SRCAA value was obtained for 18AA at 84.76, and the lowest value was obtained for 18AA- IV at 75.69. The order of the SRCCA values for the formulations was 18AA > 18AA-I > 18AA-V > 18AA-II_original research_ = 18AA-II > 18AA-IV.

The closeness of 18AA-II_original research_ and five formulations of amino acids for injection to the standard protein mode.

Table 5 shows that compared with egg protein, the highest closeness value was obtained for 18AA, 18AA-II_original research_, 18AA-II, 18AA-I at 0.94, and the lowest value was obtained for 18AA-V at 0.91. The order of the closeness values for the formulations was 18AA = 18AA-I = 18AA-II_original research_ = 18AA-II > 18AA-V > 18AA-IV.

Table 5 shows that compared with FAO/WHO protein standard mode, the highest closeness value was obtained for 18AA-I at 0.91, and the lowest value was obtained for 18AA- IVand 18AA at 0.85. The order of the closeness values for the formulations was 18AA-I > 18AA-II_original research_ = 18AA-II > 18AA-V > 18AA-IV = 18AA.

Table 6 shows the content, price, and price/content ratio of the formulation of compound amino acids for injection from different manufacturers.

**TABLE 6:** The amino acid content (g), price (yuan), and each bottle of amino acid (250 ml) from 28 manufacturers.

Manufacturer	amino acid content(g)	Price(yuan)	price/content ratio	Manufacturer	amino acid content(g)	price	price/content ratio
**18AA**				**18AA-I**			
1-18AA	12.50	11.73	0.94	**1**7-18AA-I	17.50	15.50	0.89
2-18AA	12.50	117.00	9.36	**18AA-II**(Original research)	21.25	22.78	1.07
3-18AA	12.50	100.70	8.06	**18AA-II**			
4-18AA	12.50	8.40	0.67	18-18AA-II	21.25	23.21	1.09
5-18AA	12.50	3.98	0.31	19-18AA-II	21.25	21.00	0.99
6-18AA	12.50	2.94	0.24	20-18AA-II	21.25	26.80	1.26
7-18AA	12.50	34.00	2.72	**18AA-IV**			
8-18AA	12.50	5.80	0.46	21-18AA-IV	8.70	39.00	4.48
19-18AA	12.50	10.50	0.84	22-18AA-IV	8.70	35.00	4.02
10-18AA	12.50	4.00	0.32	**18AA-V**			
11-18AA	12.50	8.40	0.67	23-18AA- V	8.70	41.00	4.71
12-18AA	12.50	10.50	0.84	24-18AA- V	8.70	39.00	4.48
13-18AA	12.50	9.77	0.78	25-18AA- V	8.70	35.00	4.02
14-18AA	12.50	7.67	0.61	26-18AA- V	8.70	45.00	5.17
15-18AA	12.50	10.20	0.82	27-18AA- V	8.70	49.00	5.63
16-18AA	12.50	10.70	0.86				

*The bold terms “18AA, 18AA-I, 18AA-II, 18AA-IV, 18AA-V, 18AA-II (original research)” are the amino acids of 5 varieties and the original varieties of amino acids, which have appeared in many places in the article. The “1-18AA, 2-18AA, 27-18AA-V” means there are 27 manufacturers.*

Table 6 shows that, among the 27 manufacturers, 12 had a price/content ratio higher than that of 18AA-II_original research_, with 2-18AA as the highest at 8.75 times that of 18AA-II_original research_. Fifteen had a price/content ratio lower than that of 18AA-II_original research_, with 11-18AA as the lowest at 4.56 times that of 18AA-II_original research._

## Discussion

In this study, by comparing the safety, of the original research manufacturers with that of the generic manufacturers, the order of safety was 18AA-II_original research_ = 18AA-II > 18AA-I > 18AA = 18AA-V > 18AA-IV. Whether amino acids can be better synthesized, the total EAA should be considered in addition to the content of a certain EAA. Through comprehensive evaluation of the first limiting amino, EAAI, SRCAA, and closeness to the EAA of the standard protein, we found that the overall effectiveness order was 18AA-II_original research_ = 18AA-II>18AA > 18AA-I>18AA-IV>18AA-V.

The emergence and development of compound amino acids for injection make it possible for patients who cannot eat to survive and prolong their life (21). Proteins are the key nutrient for survival (22) and the balance of amino acids is very important for protein synthesis in patients with normal liver and kidney function, therefore, rational selection of drugs is particularly important. Drugs for clinical application are a special commodity that should be safe, nutritional quality, and economic (23).

Moreover, among the 27 manufacturers, 12 manufacturers had a price/content ratio higher than that of 18AA-II original research manufacturers, and 15 manufacturers had a price/content ratio lower than original research manufacturers. The economy of one manufacturer of 18AA-II was slightly superior, and that of two manufacturers was slightly inferior. The only manufacturer 18AA-I had a slight advantage in the economy. 13 manufacturers had a slight advantage and 3 manufacturers had a slight disadvantage in the economy of 18AA. The 18AA-IV and 18AA-V were the most expensive from all manufacturers. Based on the above comprehensive research, when clinicians chose balanced amino acid, the 18AA-II_original research_ and 18AA-II with high safety and effectiveness should be considered first, and manufacturers with lower price/content ratios can be preferred. Secondly, 18AA with better effectiveness or 18AA-I with better safety should be selected, meanwhile, manufacturers with lower price/content ratio should be preferred. 18AA-IV or 18AA-V with poor safety, efficacy, and economy, are not recommended.

## Conclusion

This research provided a reference for drug selection of medical institutions and physicians, which not only saved costs for national medical insurance and patients but also helped patients better synthesized protein and recovered from hospital sooner.

## Data Availability Statement

The original contributions presented in the study are included in the article/supplementary material, further inquiries can be directed to the corresponding author/s.

## Author Contributions

YS, HS, JLiu, JLin, and LF carried out most of the experiment, draft, design, data analysis, prepared and wrote the manuscript. All authors read and approved the final manuscript.

## Conflict of Interest

The authors declare that the research was conducted in the absence of any commercial or financial relationships that could be construed as a potential conflict of interest.

## Publisher’s Note

All claims expressed in this article are solely those of the authors and do not necessarily represent those of their affiliated organizations, or those of the publisher, the editors and the reviewers. Any product that may be evaluated in this article, or claim that may be made by its manufacturer, is not guaranteed or endorsed by the publisher.

## References

[1] Brosnan J Rooyackers O. The importance of amino acids as independent metabolites, signalling molecules and as building blocks for protein. *Curr Opin Clin Nutr Metab Care.* (2012) 15:47–8. 10.1097/MCO.0b013e32834de431 22123615

[2] Ong XS Sultana R Tan JW Tan QX Wong JSM Chia CS The role of total parenteral nutrition in patients with peritoneal carcinomatosis: a systematic review and meta-analysis. *Cancers.* (2021) 13:4156. 10.3390/cancers13164156 34439309 PMC8393754

[3] Iacone R Scanzano C Santarpia L Cioffi I Contaldo F Pasanisi F. Macronutrients in parenteral nutrition: amino acids. *Nutrients.* (2020) 12:772. 10.3390/nu12030772 32183395 PMC7146427

[4] Watford M Wu G. Protein. *Adv Nutr.* (2018) 9:651–3. 10.1093/advances/nmy027 30060014 PMC6140426

[5] Hoffer LJ. Parenteral nutrition: amino acids. *Nutrients.* (2017) 9:257. 10.3390/nu9030257 28287411 PMC5372920

[6] Zhen X Sun X Dong H. Health technology assessment and its use in drug policies in China. *Value Health Reg Issues.* (2018) 15:138–48. 10.1016/j.vhri.2018.01.010 29729645

[7] Alshammari TM. Drug safety: the concept, inception and its importance in patients’ health. *Saudi Pharm J.* (2016) 24:405–12. 10.1016/j.jsps.2014.04.008 27330371 PMC4908051

[8] Bozzetti F. Parenteral nutrition. *Nutrition.* (2019) 66:101–7. 10.1016/j.nut.2019.03.013 31254948

[9] Wu G. Amino acids: metabolism, functions, and nutrition. *Amino Acids.* (2009) 37:1–17. 10.1007/s00726-009-0269-0 19301095

[10] Iacone R Scanzano C Santarpia L Alfonsi L Marra M Pagano MC Essential amino acid profile in parenteral nutrition mixtures: does it meet needs?. *Nutrients.* (2018) 10:1937. 10.3390/nu10121937 30563270 PMC6316548

[11] Yarandi SS Zhao VM Hebbar G Ziegler TR. Amino acid composition in parenteral nutrition: what is the evidence?. *CurrOpin Clin NutrMetab Care.* (2011) 14:75–82. 10.1097/MCO.0b013e328341235a 21076291 PMC3071792

[12] Eum S Ock M Lee S Kim H. Adverse events and concurrent medications associated with parenteral nutrition use. *Basic Clin PharmacolToxicol.* (2019) 124:154–62. 10.1111/bcpt.13116 30133153

[13] Christian VJ Tallar M Walia CLS Sieracki R Goday PS. systematic review of hypersensitivity to parenteral nutrition. *JPEN J Parenter Enteral Nutr.* (2018) 42:1222–9. 10.1002/jpen.1169 29761928

[14] Huston RK Baxter LM Larrabee PB. Neonatal parenteral nutrition hypersensitivity: a case report implicating bisulfite sensitivity in a newborn infant. *JPEN J Parenter Enteral Nutr.* (2009) 33:691–3. 10.1177/0148607109347643 19892903

[15] Vally H Misso NL. Adverse reactions to the sulphite additives. *Gastroenterol Hepatol Bed Bench.* (2012) 5:16–23. 24834193 PMC4017440

[16] Bobka MS. The 21 CFR (Code of Federal Regulations) online database: food and drug administration regulations full-text. *Med Ref Serv Q.* (1993) 12:7–15. 10.1300/J115V12N04_02 10126251

[17] Timbo B Koehler KM Wolyniak C Klontz KC. Sulfites–a food and drug administration review of recalls and reported adverse events. *J Food Prot.* (2004) 67:1806–11. 10.4315/0362-028x-67.8.1806 15330554

[18] Dong H Liu J Zeng X Bai W Yu L. Enzymatic hydrolysis pretreatment for enhancing the protein solubility and physicochemical quality of Cordyceps militaris chicken soup. *Food Sci Nutr.* (2020) 8:2436–44. 10.1002/fsn3.1533 32405400 PMC7215234

[19] Jiang Y Nie WJ. Chemical properties in fruits of mulberry species from the Xinjiang province of China. *Food Chem.* (2015) 174:460–6. 10.1016/j.foodchem.2014.11.083 25529706

[20] Tian H Guo G Fu X Yao Y Yuan L Xiang A. Fabrication, properties and applications of soy-protein-based materials: a review. *Int J Biol Macromol.* (2018) 120:475–90. 10.1016/j.ijbiomac.2018.08.110 30145158

[21] Zhang P Ma G Wang C Lu H Li S Xie Y Effect of irrigation and nitrogen application on grain amino acid composition and protein quality in winter wheat. *PLoS One.* (2017) 12:e0178494. 10.1371/journal.pone.0178494 28594830 PMC5464558

[22] Sibian MS Saxena DC Riar CS. Effect of germination on chemical, functional and nutritional characteristics of wheat, brown rice and triticale: a comparative study. *J Sci Food Agric.* (2017) 97:4643–51. 10.1002/jsfa.8336 28370158

[23] Yang H Li X Gao J Tong P Yang A Chen H. Germination-assisted enzymatic hydrolysis can improve the quality of soybean protein. *J Food Sci.* (2017) 82:1814–9. 10.1111/1750-3841.13782 28631814

